# Boosting executive functions and math abilities in adolescents with dyscalculia: the combined effect of prismatic adaptation and cognitive training

**DOI:** 10.3389/fpsyg.2025.1669090

**Published:** 2026-01-12

**Authors:** Lilian Zotti, Lauro Quadrana, Giulia Conte, Eleonora Sbardella, James Dawe, Agnese Di Garbo, Lina Pezzuti, Massimiliamo Oliveri

**Affiliations:** 1 Department of Dynamic and Clinical Psychology and Health, Sapienza University of Rome, Rome, Italy; 2 Department of Human Neurosciences, Sapienza University of Rome, Rome, Italy; 3 Department of Developmental and Social Psychology, Sapienza University of Rome, Rome, Italy; 4 Restorative Neurotechnologies Srl, Palermo, Italy; 5 Department of Biomedicine, Neurosciences and Advanced Diagnostics, University of Palermo, Palermo, Italy

**Keywords:** adolescence, cognitive training, dyscalculia, higher-order cognitive processes, prismatic adaptation

## Abstract

Developmental dyscalculia (DD) is a neurodevelopmental disorder characterized by both functional and structural brain modifications. Individuals with DD exhibit impairments in higher-order cognitive processes. Timely intervention is crucial, as difficulties in mathematical learning can impact academic achievement, contribute to behavioral problems, and increase the risk of school dropout. This study aims to explore whether targeting higher-order cognitive processes could partially remediate developmental dyscalculia, with pre-registered primary outcomes including numerical facts, mental calculation speed, and mental calculation accuracy (ISRCTN, ISRCTN15190285). We conducted a single-center, two-arm, randomized controlled trial. Seventy adolescents aged 13–17 years, who had recently received a first diagnosis of developmental dyscalculia, were randomly assigned to either the intervention or waitlist group. The intervention leveraged the synergy between an initial stimulation phase—utilizing prismatic adaptation, a bottom-up visuomotor rehabilitation technique, to activate neural activity in brain regions involved in cognitive processing—followed by a targeted (top-down) cognitive training phase designed to strengthen the specific skills underlying mathematical deficits. Group differences over time were examined using a general linear model with two groups (treatment vs. control) and two-time points (pre- and post-treatment) for each outcome variable. Significant increases were observed in the working memory index (between group change = 16.51 (CI = 13.59–19.44); *p* < 0.001, *η*^2^ = 0.66), processing speed index (between group change = 12.40 (CI = 9.73–15.70); *p* < 0.001, *η*^2^ = 0.58), mental calculation speed (between group change = 0.74 (CI = 0.55–0.93); *p* < 0.001, *η*^2^ = 0.46), mental calculation accuracy (between group change = 0.43 (CI = 0.14–0.71); *p* < 0.001, *η*^2^ = 0.17), and numerical fact retrieval (between group change = 14.07 (CI = 8.58–19.56); *p* < 0.001, *η*^2^ = 0.31). These findings suggest that combining neurostimulation via prismatic adaptation with targeted cognitive training using serious games enhances both mathematical accuracy and response speed, as well as executive functions, attention, inhibition, and cognitive processing. Prismatic adaptation appears to enhance activity in brain regions that are subsequently reinforced by cognitive training. This innovative approach could have important implications for the early remediation of core neuropsychological deficits in dyscalculia.

## 1 Introduction

Developmental dyscalculia (DD) is a specific learning disorder characterized by persistent difficulties in numerical understanding despite normal cognitive abilities and schooling opportunities (Agostini et al., 2022; American Psychiatric Association, 2022; Mishra and Khan, 2023). To diagnose DD, symptoms must persist for at least 6 months and create impairments in multiple areas of life, such as social, academic, and work settings (American Psychiatric Association, 2022; Mishra and Khan, 2023). DD is frequently comorbid with a range of complications that warrant detailed characterization, including Attention-Deficit/Hyperactivity Disorder (ADHD) and other neurodevelopmental conditions, as well as anxiety and mood disorders (American Psychiatric Association, 2022; Price and Ansari, 2013; Saga et al., 2022). Following DSM-5-TR criteria, all types of specific learning disorders have a prevalence of 5–15% in the school population worldwide (American Psychiatric Association, 2022) with estimates of DD around 1.3–13.8% (Morsanyi et al., 2018). The evidence points to the absence of a consistent gender predominance in DD (Cantlon, 2022; Devine et al., 2013; Giofrè et al., 2022; Luoni et al., 2023).

Despite the high prevalence of DD, the lack of knowledge about the disorder has led to difficulties in diagnosing and offering effective treatments (Palaniswamy et al., 2023). Research in this field is in its infancy compared to other areas of learning and there is still limited understanding of the causes underlying the neurocognitive deficits commonly found in the disorder (Mishra and Khan, 2023). From an etiological point of view, DD has been increasingly conceived as a neurodevelopmental condition primarily characterized by a neurological basis, originally described as involving brain regions responsible for fundamental mathematical abilities while other mental functions remain unaffected (Kosc, 1974). Although genetic and environmental factors have been proposed as potential contributors (Butterworth and Laurillard, 2010), evidence supporting their role is still emerging and less substantial compared to that available for other neurodevelopmental disorders. The neurobiology of DD involves both functional and structural modifications in specific brain areas (Kuhn et al., 2016) such as the intraparietal sulcus (IPS) of the parietal lobe (Mishra and Khan, 2023). From a neuropsychological point of view, different underlying impairments have been identified, including defective number sense, working memory deficit (Goffin and Ansari, 2016), or visuospatial difficulties (Saga et al., 2022; McCaskey et al., 2017).

Two main theoretical accounts are commonly used to explain the symptoms of DD, one specific and one general (Bellon et al., 2016; Mazzocco et al., 2011). The specific account is the ‘core deficit hypothesis’, claiming that the deficits in mathematical reasoning arise from the impairment of basic numerical abilities such as the number sense (Mazzocco et al., 2011; Butterworth, 2005; Wilkey, 2023), i.e., the preverbal ability to represent numerosity (Dehaene and Cohen, 1997). Conversely, according to the general approach or ‘domain general hypothesis’, the numeracy deficit in DD is due to an impairment in multiple cognitive functions, including memory and visuo-spatial attention (Mishra and Khan, 2023; Szucs et al., 2013). Deficiencies in these higher-order cognitive processes would subsequently contribute to difficulties in the skills required for mathematical abilities (Mishra and Khan, 2023). The domain-general hypothesis is supported by several studies in children with DD showing defective executive functions and attention. *Working memory* (Stefanelli and Alloway, 2020) has been extensively investigated in relationship to mathematical abilities as it is crucial for calculation to both remember and, subsequently, retrieve a sequence of numbers (Andersson and Östergren, 2012; Layes et al., 2018). An impairment in this cognitive function may result in immature strategies to solve mathematical tasks and problems (Agostini et al., 2022; Szucs et al., 2013; DeStefano and LeFevre, 2004) and there is robust evidence of worse working memory in children with DD (McLean and Hitch, 1999; Geary et al., 1999; Geary et al., 1991; Passolunghi and Siegel, 2004). Other lines of research support the presence of *visuospatial attention deficits* in DD (Geary, 2004). Visuospatial processing appears as a critical component of arithmetic processing (Price and Ansari, 2013) because when we think about numbers we organize them in space along a mental ‘number line’ (Dehaene et al., 1993; Ito and Hatta, 2004); and this mapping is independent from formal mathematical instruction (De Hevia and Spelke, 2009). In line with this view, different studies suggest that visuospatial abilities can predict mathematics performance in children (Szucs et al., 2013; Tosto et al., 2014). *Processing speed* is also a crucial cognitive function for mathematical skills, as it facilitates basic tasks such as counting and number decoding (Agostini et al., 2022). Processing speed is closely linked to store information allowing children with higher processing speed to retain more information in mind so it is easier to associate options with outcomes (Agostini et al., 2022). From a developmental perspective, processing speed is considered a key factor in age-related differences in numerical skills (Peng et al., 2018). Indeed, research has observed that children with mathematical difficulties often exhibit significantly lower processing speed (Cirino et al., 2015; Fuchs et al., 2006; Fuchs et al., 2008; Peng et al., 2018). Pedagogical interventions are one of the possible interventions for dyscalculia, they often involve collaboration between tutors, teachers, and parents to provide continuous feedback and support (Palaniswamy et al., 2023; Baker et al., 2002). They aimed to adapt the student’s learning style, addressing specific deficits associated with dyscalculia (Delgado et al., 2019). The goal is to implement targeted strategies that enhance the educational process by involving the classroom, the teacher, the family, and the various learning environments (Delgado et al., 2019). However, treatments at the pedagogical level may be limited since they do not directly act on all the cognitive factors underlying developmental dyscalculia. For this reason, increasing evidence highlights the central role of working memory, visuospatial attention, and processing speed in mathematical performance, suggesting these domains as promising targets for remediation.

Therefore, accumulating evidence points out that working memory, visuospatial attention, and processing speed are relevant for mathematical skills and may represent valuable targets in remediating DD. Cognitive rehabilitation is a therapeutic approach aimed at enhancing specific cognitive functions through various tools and techniques, including training of working memory and executive functions (Arnsten, 2009). By inducing neuroplastic mechanisms in brain areas involved in certain cognitive functions, cognitive rehabilitation can positively influence deficient cognitive abilities associated with DD and, subsequently, improve mathematical performance (Habibi Khouzani et al., 2023). Treatments involving targeted stimulation of certain brain areas (Bigler, 2004) have been developed also in adolescents (Lazzaro et al., 2023). Numerous studies (Lazzaro et al., 2023; Bertoni et al., 2023) have demonstrated the efficacy of neuromodulation intervention (tRNS, tACS, tDCS) for the treatment of neurodevelopmental disorders and specifically, dyscalculia (Mohammadi Molod et al., 2023; Moret et al., 2019). Studies in this area are still recent, but there are promising results regarding the modulation of cognitive processing (Brunoni and Vanderhasselt, 2014), numerical skills (Schroeder et al., 2017; Van Bueren et al., 2021), and learning itself (Simonsmeier et al., 2022).

Within this emerging field, an increasing number of interventions—particularly for children—use child-friendly, often computerized cognitive rehabilitation tools, such as “serious games,” to enhance engagement and accessibility (Alipanah et al., 2022). These game-based platforms are designed to stimulate learning by maintaining high levels of motivation and fostering a sense of independence and control over the tasks performed (Vogel et al., 2006; Magnani et al., 2014). Some rehabilitation programs particularly rely on the use of ‘serious games’ in digital therapies via PCs or tablets (Wouters et al., 2013). These games are interactive (Vogel et al., 2006), rely on a set of rules (Garris et al., 2002), and most importantly, almost always have a goal to achieve (Malone, 1981). Moreover, games generally provide feedback through a score so that children can check their own progress (Garris et al., 2002). Serious games aim not to entertain but rather to use entertainment to train and educate (Zyda, 2005). Thus, gamification of cognitive training may represent a promising strategy to enhance learning and engagement, particularly in children and adolescents (Gray and MacBlain, 2015). Gamified cognitive training has been shown to improve mathematical performance more effectively than non-cognitive game-based interventions (Lazzaro et al., 2023).

Despite growing evidence for the efficacy of these approaches, very few studies have investigated cognitive or game-based rehabilitation specifically in adolescents with dyscalculia, and research targeting this age group remains limited. The present study addresses this gap by evaluating whether targeting higher-order cognitive processes could partially remediate developmental dyscalculia, providing novel evidence on intervention strategies tailored to adolescents—a population that has received significantly less attention in previous research.

Similarly, prismatic adaptation (PA) is a promising technique for modulating cognitive functions through a bottom-up approach. PA is a visuomotor rehabilitation technique characterized by the use of prismatic goggles that shift the perceived visual field either to the left or right compared to the actual position of objects (Magnani et al., 2014). Originally developed to treat patients with spatial neglect (Rossetti et al., 1998), its application has expanded to various contexts over time, highlighting its versatility as a neurorehabilitation tool. Neuroimaging research has highlighted that PA not only corrects visuomotor errors but also induces changes in brain areas involved in higher-level cognitive functions (Michel, 2016; Bracco et al., 2018; Clarke and Crottaz-Herbette, 2016) while other research has demonstrated improvements in attention (Wilf et al., 2019), spatial and temporal cognition, visuospatial abilities (Pisella et al., 2006), language (Turriziani et al., 2021), short and long term memory (Turriziani et al., 2024), following PA. Moreover, PA exposure—whether brief or prolonged—can contribute to long-term reorganization of brain areas like the cerebellum and posterior parietal cortex (Pisella et al., 2006). Lastly, in another study from our group, rightward PA combined with computerized cognitive rehabilitation improved reading abilities in adolescents with dyslexia (Conte et al., 2024), another specific learning disorder which is often comorbid with DD and shows overlapping cognitive underpinnings (Moll et al., 2021).

Our proposed intervention implements Prismatic Adaptation (PA) and cognitive training through the MindLenses® software to enhance neuroplasticity and cognitive functions in adolescents with dyscalculia and executive function deficits. This two-phase approach—prismatic activation followed by serious games—aims to improve both cognitive performance and therapy adherence, as serious games have been shown to increase motivation and compliance in rehabilitation (Craven and Groom, 2015).

Building on previous evidence suggesting that executive functions and attention are key predictors of mathematical abilities and are often differently impaired in individuals with DD, the present study explores whether targeting these higher-order cognitive processes could partially remediate DD. Specifically, we adopted a combined intervention approach for adolescents with DD, integrating PA as a bottom-up visuomotor rehabilitation technique together with a top-down cognitive training aimed at enhancing working memory and processing speed. Based on these premises, we hypothesized two main outcomes: (i) a significant improvement in mathematical performance in the active intervention group compared to the control group, and (ii) a consistent modulation of working memory and processing speed, reflecting a synergistic effect of the combined intervention.

## 2 Methods

We conducted a quantitative study using a pseudo-randomized allocation to the treatment and waitlist groups without blinding, balanced for biological sex and age. The study design, sample selection, inclusion and exclusion criteria, management of comorbidities, assessor training, and instrument validity are detailed in the following subsections.

### 2.1 Study design

All study procedures were approved by the Policlinico Umberto I Ethical Committee and were carried out in accordance with the Code of Ethics of the World Medical Association (Declaration of Helsinki). Parents/guardians and youth participants provided written consent and written and verbal assent, respectively. The study has been publicly registered on 21/11/2023 on the ISRCTN registry (clinical trial number: ISRCTN15190285).

This study is designed as a single-center randomized controlled trial, consisting of 10 weeks of cognitive training followed by a 3-month follow-up period. Participants will be recruited from the Child and Adolescent Neuropsychiatry Unit at Policlinico Umberto I in Rome, which provides both screening services and neuromodulation therapy. The trial will include two arms: an experimental group and a waitlist control group. The randomization wasn’t blind; all participants could understand whether they were in the treatment protocol or were waiting for follow-up time. After the 3 months, participants in the waitlist control group will be offered the same intervention program.

When a child presented to the clinic and met the inclusion/exclusion criteria, participation in the study was proposed to the parents. Both the parents and the child received information about the study and the characteristics of the intervention, and informed consent was obtained. Upon agreement, 70 adolescents were enrolled and stratified into two groups: a treatment group (*n* = 35), which received a combination of rightward prism adaptation (rPA) and cognitive training, and a waitlist control group (*n* = 35). The choice of rightward prism adaptation was made in the attempt to modulate right hemispheric circuits, critically involved in both visuospatial attention and representational abilities, and in core numerical/arithmetical processes (Benavides-Varela et al., 2017). Participants were randomly assigned using a stratified randomization method to balance sex (male, female) and age (13–14 years, 15–17 years). Stratification yielded four possible blocks (e.g., female, 13–14 years), ensuring balanced group allocation for baseline characteristics.

We test participants two times, before the treatment (≤1 week pre-intervention) and at the end of it (≤1 week after the final session). All adolescents completed the tasks under standardized viewing and luminance conditions, seated comfortably at a desk in a quiet room with the research assistant (Table 1).

**Table 1:** Study protocol with pre-treatment (T0) and post-treatment (T1) assessments.

Group	Age		Biological sex *nF* (*F*%)	Measures	T0	Intervention	T1
	M (SD)	Min–max					
Control	15.14 (0.81)	13–17	18 (51.4%)	Working memory index	✓	Waitlist	✓
				Processing speed index	✓		✓
				Numerical fact	✓		✓
				Mental calculation speed	✓		✓
				Mental calculation accuracy	✓		✓
Experimental	15.39 (0.75)	14–17	19 (57.6%)	Working memory index	✓	PA + cognitive training	✓
				Processing speed index	✓		✓
				Numerical facts	✓		✓
				Mental calculation speed	✓		✓
				Mental calculation accuracy	✓		✓

*M*, mean; SD, standard deviation; *nF*, number of females; *F*%, percentage of females.

The treatment protocol comprised 10 weekly sessions, each lasting 30 min (5 min for rPA, 25 min for cognitive training). No adverse events were reported during the trial.

Waitlist participants were informed at baseline that they would receive the intervention at the end of the study period; however, their post-treatment outcomes were excluded from the analysis to minimize confounding variables.

### 2.2 Participants

A total of 70 participants aged 13–17 years old (mean = 15.8; standard deviation = 0.7), of which 32 males and 38 females receiving a first diagnosis of developmental dyscalculia (DD) were recruited from the Learning Disorders specialty clinic at the Child and Adolescent Neuropsychiatry Division, Sapienza University of Rome. All subjects were right-handed and had normal or corrected-to-normal vision. Diagnosis of DD was based on neuropsychological evaluation, according to DMS-5-TR criteria, and conformed with the Italian criteria, i.e., having a score below two standard deviations (−2 SD) on assessments designed to evaluate numerical facts, as well as speed and accuracy in mental calculation. Participants were consecutively recruited from September 2023 to December 2023, and none had previously undergone treatments specifically targeted at improving calculation skills. *A priori* power analysis using standard values (small effect size *f* = 0.25, *r* = 0.5, power = 80%, G*Power v3.1.9) suggests a total sample size of 34 individuals (*n* = 17 per group).

Inclusion criteria were as follows: (a) diagnosis of dyscalculia; (b) age between 13 and 17 years old; (c) total IQ above the low average range (≥80), as confirmed on the Wechsler intelligence scale for children—fourth edition (WISC-IV); (d) either Working Memory Index (WMI) or Processing Speed Index (PSI) below the low average range (<80), or both.

The following exclusion criteria were considered: (a) the presence of other neurodevelopmental disorders, particularly of attention-deficit/hyperactivity disorder (ADHD); (b) any major comorbid psychiatric disorder such as schizophrenia, bipolar disorder, or major depression disorder; (c) a diagnosis of active epilepsy; (d) physical disabilities that could impair the use of the study instruments. Exclusion criteria (a) and (b) were assessed through the Kaufman Schedule for Affective Disorders and Schizophrenia (KSADS-PL) clinical interview conducted separately with the patient and caregivers. Exclusion criteria (c) and (d) were ruled out through accurate history taking by a child psychiatrist experienced in neuromotor and neurological disorders of childhood.

### 2.3 Intervention protocol

Participants in the intervention group underwent 10 weekly sessions combining rightward prism adaptation (rPA) with cognitive training, delivered via the MindLenses software.^1^ The treatment protocol comprised two sequential phases: a visuomotor adaptation task utilizing prismatic goggles, followed by tablet-based cognitive training. Sessions were conducted in a well-lit, distraction-minimized room. Verbal task instructions were provided at the start of each session by trained experimenters.

**Figure 1:**
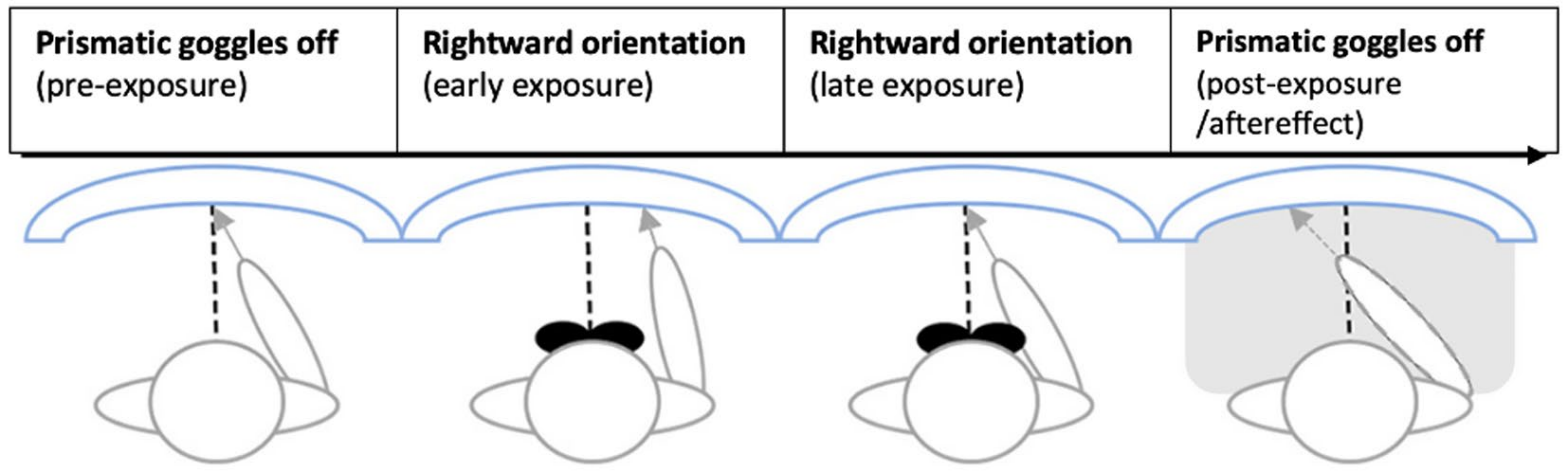
PA setup and timeline—modified with permission from (Bracco et al., 2018). Participants point to targets on a tablet screen (here depicted as curved surface, although the screen was flat). The dashed line marks the position on the screen of the visual target, while the arrow the pointing spot indicated by the participant. Pre-exposure (prismatic goggles off) involves pointing in free viewing conditions (both pointing movements and targets visible). During Exposure, participants wear the googles (rightward orientation) during free viewing pointing (exposure, goggles on). In post-exposure adaptation is then tested immediately after exposure with blinded pointing to targets (aftereffect).

In the first phase of each session, participants performed a visuomotor adaptation task wearing 20-diopter rightward-deviating prismatic goggles. They were instructed to point at randomly positioned targets on a tablet screen using their right index finger. The task lasted 3 min, comprising approximately 150 pointing movements, and was repeated three times: initially without the lenses to familiarize participants with the task, then with the lenses to induce visuomotor adaptation and brain stimulation, and finally without the lenses to recalibrate visuomotor coordination. The prismatic goggles initially induced a 20° rightward visual displacement, resulting in systematic pointing errors. Over successive trials, participants spontaneously adjusted, achieving accurate target pointing through visuomotor recalibration (see Figure 1).

Immediately after the rPA phase, participants engaged in seven tablet-based “serious games” designed to target attention, inhibition, working memory, and problem-solving skills. These games integrate simulation, entertainment, and learning to enhance cognitive abilities by simulating specific activities.

### 2.4 Instruments

#### 2.4.1 Wechsler intelligence scale for children-IV (WISC-IV)

The Italian adaptation of the Wechsler Intelligence Scale for Children—Fourth Edition (WISC-IV) (Orsini et al., 2012) was utilized to assess cognitive abilities, including the full IQ. The WISC-IV comprises 10 core subtests, organized to generate four primary index scores: Verbal Comprehension, Perceptual Reasoning, Working Memory, and Processing Speed. Index scores are standardized with a mean (M) of 100 and a standard deviation (SD) of 15, based on age-specific norms. WISC-IV has great validity, reliability, and good test–retest validity (Orsini et al., 2012; Andrikopoulos, 2021; Pezzuti et al., 2022; Ryan et al., 2010).

We used Working Memory Index (WMI) and Processing Speed Index (PSI) as outcome measures of executive skills to reliably assess group differences before and after treatment (Giofrè and Cornoldi, 2015; Poletti, 2017), given their minimal susceptibility to learning effects upon repeated testing (Pezzuti et al., 2022). They, respectively, evaluate the ability to sustain focused attention, encode, retain, and manipulate new information for task completion, and to assess sustained attention, visual scanning, discrimination, short-term retention of unfamiliar visual stimuli, and visuomotor coordination (Orsini et al., 2012). WMI and PSI were used to accurately evaluate differences between groups pre- and post-treatment, since they show little or no learning effects at re-test overtime.

#### 2.4.2 MT-3 avanzate

An Italian test battery (Cornoldi et al., 2017) validated to diagnose specific learning disorders in reading, writing, mathematics, and comprehension. For the present study, we selected specifical subtests as primary indicators of mathematical abilities designed to assess numerical fact retrieval and mental calculation abilities.

Our outcome measures include Mental Calculation accuracy (MC accuracy) assessed through the number of correct responses, Mental Calculation speed (MC speed) assessed through the seconds taken giving the answer, and Numerical Fact Retrieval (NF) to assess the automatization of calculation processes. Participants were instructed to perform each mental calculation aloud, responding as quickly and accurately as possible.

The manual reports an acceptable level of internal consistency for the entire numerical competence test (Cronbach’s alpha = 0.65), while the test–retest validity demonstrates a moderately high correlation (*r* = 0.53) (Cornoldi et al., 2017).

### 2.5 Data analysis

Statistical analyses were performed using IBM SPSS Statistics (version 27). The primary analyses followed an intention-to-treat (ITT) approach, including all randomized participants in the groups to which they were originally allocated. Because all 70 adolescents completed both assessments and there were no protocol deviations leading to exclusion, the ITT and per-protocol (PP) populations coincided.

Group differences over time were examined via a general linear model (GLM) with two groups (treatment vs. control) and two time points (pre- and post-treatment) for each outcome variable. A group-by-time interaction term was tested to determine whether changes in the Working Memory Index (WMI), Processing Speed Index (PSI), Mental Calculation (MC) speed, Mental Calculation (MC) accuracy, and Numerical Fact (NF) from pre- to post-treatment differed between the two groups. Biological sex and age groups were added as covariates. To control type I error inflation due to multiple comparisons across the five outcome variables, we applied a Bonferroni correction (adjusted *α* = 0.01; 0.05/5).

Additional ANOVAs were conducted to explore differences at baseline and post-treatment between the control and treatment groups, as well as within each group over time, for all variables included in the study.

Finally, we conducted post-hoc analysis to examine the interaction effect of demographic variables (age group and biological sex) using mixed GLM for repeated measures, in order to explore whether the effect of the intervention varied across sub-groups. In case of significant interactions, stratified exploratory analysis was conducted.

Effect sizes were quantified using eta squared (*η*^2^) and partial eta squared (*η*^2^). As the value of *η*^2^ depends on the number and size of other effects in the model, partial *η*^2^ was considered as practical alternative (Tabachnick and Fidell, 2019). According to Cohen (1988), *η*^2^ values of 0.01, 0.09, and 0.25 correspond to small, medium, and large effects, respectively (Cohen, 2013).

## 3 Results

### 3.1 Baseline characteristics

**Figure 2:**
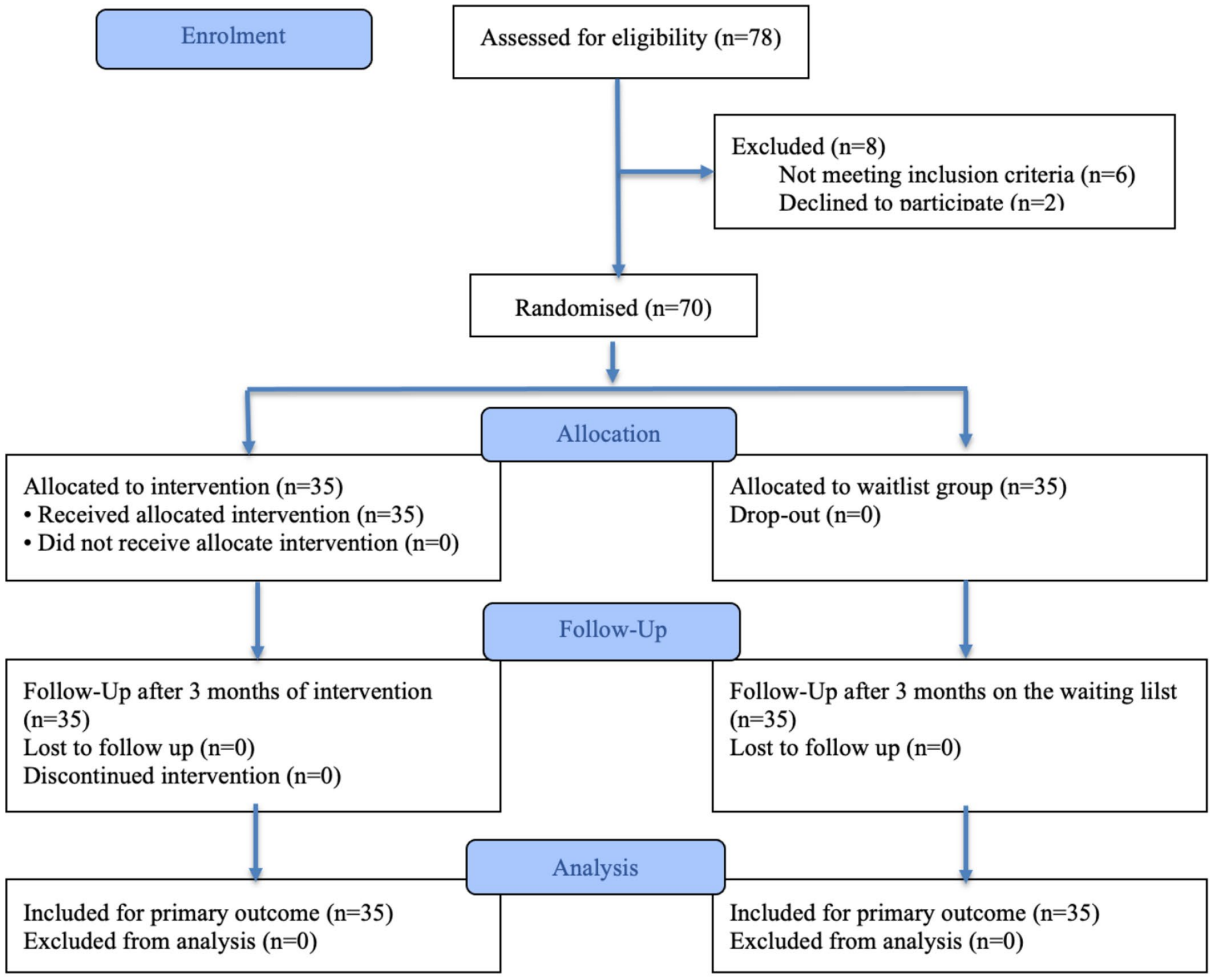
CONSORT 2025 Flow Diagram of the progress through the phases of a randomised trial of two groups.

Of the 78 individuals who were enrolled in the research, six did not meet the inclusion criteria, and 2 declined to participate. None of the 70 allocated dropped out from the study or deviated from the intended intervention, thus all completed the baseline and post-treatment assessments (Figure 2). Baseline characteristics are reported in Supplementary material 1. No significant differences were observed between groups for sex (*p* = 0.81), chronological age, full-scale IQ, WMI, PSI, or pre-treatment mathematical abilities (all *p*-values > 0.05).

### 3.2 Main effects

**Figure 3:**
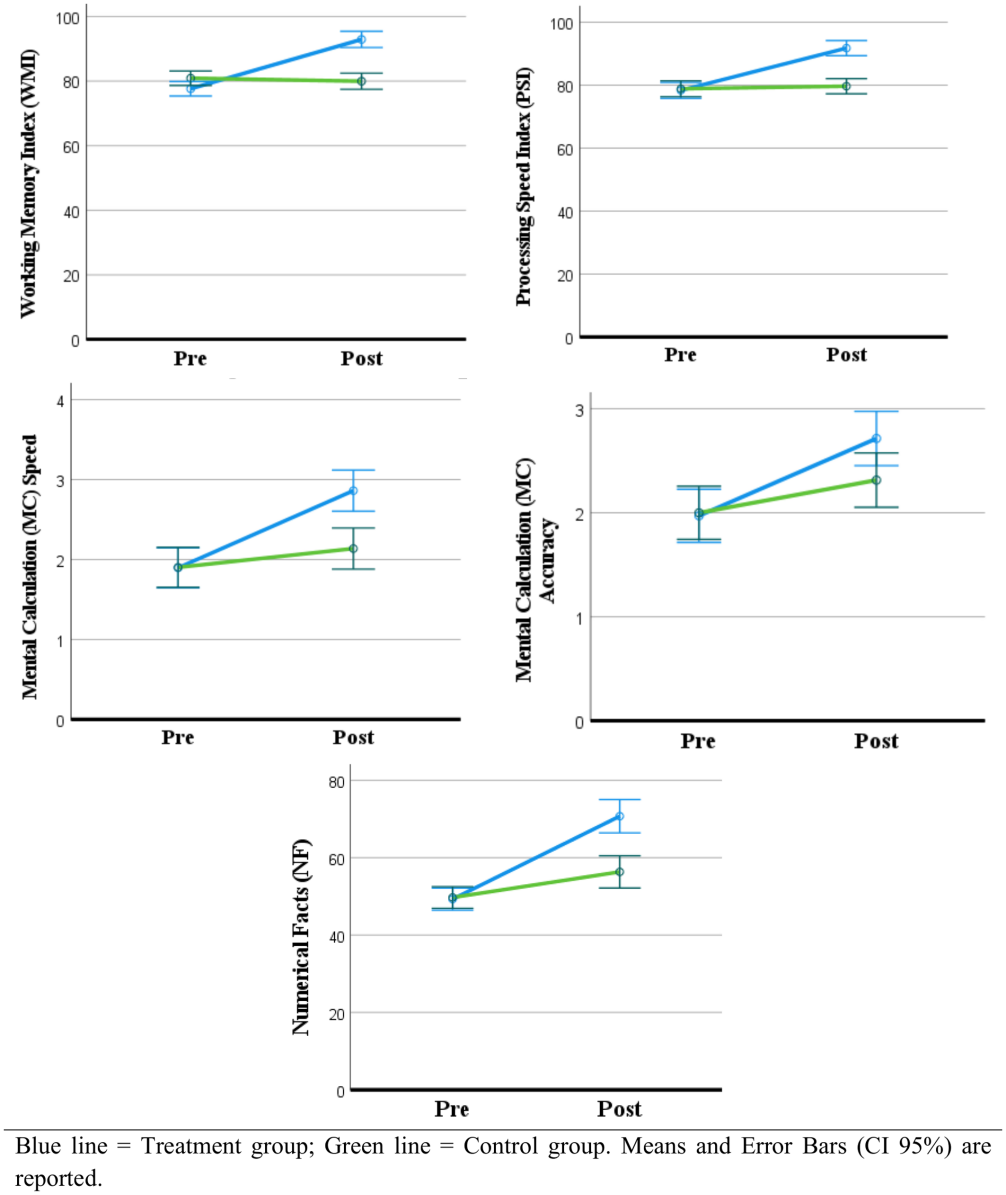
Graphical representation of the change over time between Treatment and Control.

Data from all 70 participants in the trial were included in the analyses. A 2 × 2 (PA group/Waitlist group × pre-/post-treatment) GLM with repeated measures (biological sex and age groups were added as covaries) revealed a significant effect of time on all the outcome measures (Table 2), indicating an overall increase in all outcome measures at post-treatment assessments (Figure 3). This effect was driven by a significant interaction effect for Group by Time, revealing that changes in outcome measures over time were largely explained by group membership (Table 2). Mean changes in WMI, PSI, MC speed, MC accuracy, and NF from baseline to post-test assessment between groups are displayed in Figure 3. At univariate level (Table 2), significantly better performances were found in the treatment group for all variables at group comparisons over time: WMI [*F*(1,68) = 100.853, *p* < 0.001, *η*^2^ = 0.597], PSI [*F*(1,68) = 148.226, *p* < 0.001, *η*^2^ = 0.686], MC speed [*F*(1,68) = 66.989, *p* < 0.001, *η*^2^ = 0.496], MC accuracy [*F*(1,68) = 9.021, *p* = 0.004, *η*^2^ = 0.117], NF [*F*(1,68) = 26.218, *p* < 0.001, *η*^2^ = 0.278].

**Table 2:** Results from the analysis of variance for group (treatment vs. control) by Time (pre-, post-treatment) effects for the variables included in the study.

Group	*N*	Baseline M (SD)	Post-treatment M (SD)	Δ Pre–post	Between-group change (95% CI)	GLM results
Working memory index (WMI)						
T	35	77.80 (6.87)	93.23 (8.99)	15.43	16.51 (13.59–19.44)	*F*(1,68) = 128.132, *p* < 0.001, *η*^2^ = 0.66
C	35	80.77 (6.16)	79.69 (6.05)	−1.09		
Processing speed index (PSI)						
T	35	78.37 (8.40)	91.69 (6.79)	13.31	12.40 (9.73–15.7)	*F*(1,68) = 89.537, *p* < 0.001, *η*^2^ = 0.58
C	35	78.89 (6.17)	79.23 (7.52)	0.91		
Mental calculation (MC) speed						
T	35	1.91 (0.74)	2.89 (0.72)	0.97	0.74 (0.55–0.93)	*F*(1,68) = 56.525, *p* < 0.001, *η*^2^ = 0.46
C	35	1.89 (0.72)	2.11 (0.80)	0.23		
Mental calculation (MC) accuracy						
T	35	1.97 (0.82)	2.71 (0.75)	0.74	0.43 (0.14–0.71)	*F*(1,68) = 12.675, *p* < 0.001, *η*^2^ = 0.17
C	35	2.00 (0.69)	2.31 (0.80)	0.31		
Numerical facts (NF)						
T	35	49.33 (8.12)	70.43 (10.35)	21.09	14.07 (8.58–19.56)	*F*(1,68) = 29.353, *p* < 0.001, *η*^2^ = 0.31
C	35	49.86 (8.11)	63.66 (14.26)	7.03		

*N*, number of subjects; *M*, mean; SD, standard deviation; Δ change, difference in change between treatment and control group; CI, 95% confidence intervals; GLM, general linear model; *T*, treatment group; *C*, control group; *η*^2^, partial eta squared.

Post hoc analysis showed that all outcome measures significantly increased from pre- to post-test in the treatment group with medium to large effect sizes (Table 3). Specifically there was significant increase in WMI [*F*(186,536), *p* < 0.001, *η*^2^ = 0.846], in PSI [*F*(311,348), *p* < 0.001, *η*^2^ = 0.902], MC speed [*F*(225,885), *p* < 0.001, *η*^2^ = 0.869], MC accuracy [*F*(51,766), *p* < 0.001, *η*^2^ = 0.604], NF [*F*(168,356), *p* < 0.001, *η*^2^ = 0.832]. Moreover, post hoc analysis showed a significant improvement with a small effect size in the control group from pre- to post-test (Table 3): WMI [*F*(6,555), *p* 0.015, *η*^2^ = 0.162], MC speed [*F*(8,500), *p* 0.006, *η*^2^ = 0.200], MC accuracy [*F*(10,183), *p* 0.003, *η*^2^ = 0.230], NF [*F*(10,069), *p* 0.003, *η*^2^ = 0.228].

**Table 3:** Post-hoc results for the simple effect for groups and time on the variables included in the study.

Variables	Pre vs post-treatment for control group			Pre vs post-treatment for treatment group			Control vs treatment at baseline			Control vs treatment at post treatment		
	*F*(df)	*p*	*η*^2^ ^a^	*F*(df)	*p*	*η*^2^^a^	*F*(df)	*p*	*η* ^2^ ^b^	*F*(df)	*p*	*η*^2^ ^b^
WMI	6.555 (34)	0.015	0.162	186.536 (34)	<0.001	0.846	0.000 (34)	1.000	–	54.650 (34)	<0.001	0.446
PSI	1.538 (34)	0.223	–	311.348 (34)	<0.001	0.902	0.000 (34)	1.000	–	52.856 (34)	<0.001	0.437
MC Speed	8.500 (34)	0.006	0.200	225.885 (34)	<0.001	0.869	0.000 (34)	1.000	–	18.118 (34)	<0.001	0.210
MC Accuracy	10.183 (34)	0.003	0.230	51.766 (34)	<0.001	0.604	0.025 (34)	0.875	–	4.680 (34)	0.034	0.064
NF	10.0.69 (34)	0.003	0.228	168.356 (34)	<0.001	0.832	0.074 (34)	0.786	–	20.662 (34)	<0.001	0.233

WMI, working memory index; PSI, processing speed index; MC, mental calculation; NF, numerical fact.

a partial eta squared.

b eta squared.

The simple effect between the groups at baseline shows no significant differences for any variable while there are significant differences in each variable in the comparison between the groups after treatment.

### 3.3 Interaction effects

To analyze the possible effects of biological sex and age groups (13–15 vs. 16–17), two additional mixed GLMs for repeated measures were conducted, including these two demographic variables as between-participants factors. All results were non-significant (see Supplementary material 1).

## 4 Discussion

To our knowledge, this is the first study to evaluate the efficacy of PA coupled with cognitive training for the treatment of dyscalculia in adolescents. Our results demonstrate that 10 weekly sessions of the proposed intervention significantly improved all mathematical performance metrics. Furthermore, significant improvements in working memory and speed processing abilities were induced, suggesting that the treatment allows modulation of cognitive functions relevant for mathematical performance (Stefanelli and Alloway, 2020).

Treatment approaches for dyscalculia vary based on the underlying deficit theory. If a general cognitive deficit is identified, interventions should focus on strengthening weaker cognitive domains, whereas a specific deficit would require targeted training in mathematical skills (Alipanah et al., 2022). Given the multifactorial etiology of dyscalculia, various remediation strategies have been developed and studied, including pedagogical interventions, game-based therapy, neuromodulation of relevant brain areas, and cognitive rehabilitation approaches.

While the core-specific theory of DD has dominated neuroscience research for the last decades, more recent behavioral research has stressed the role played by other cognitive functions in mathematical development (Agostini et al., 2022; Szucs et al., 2013; Donlan et al., 2007; Geary et al., 2007). Moreover, growing evidence suggests that dyscalculia is not solely rooted in numerical deficits but rather emerges from the complex interplay between domain-specific and domain-general cognitive processes. The study by Filippo and Zoccolotti (2018) demonstrates for the first time that, as previously observed in reading, numerical performance can also be explained by a global factor. This perspective underscores the need for comprehensive assessment protocols that extend beyond numerical abilities to include broader cognitive functions such as working memory, processing speed, and executive functioning.

A multidimensional evaluation is crucial for tailoring intervention strategies that address both the core deficits and the associated cognitive weaknesses, ultimately enhancing treatment efficacy (Agostini et al., 2022). In this regard, the intervention proposed in our study may improve overall cognitive processing efficiency and the complex mechanisms involved in mathematical abilities.

Unlike other visual stimulation techniques that require prolonged exposure, PA has a rapid onset of effects. Significant improvements can be observed after only a few minutes of prism exposure (Kerkhoff et al., 2006; Pizzamiglio et al., 2006) and short-term interventions of 2 weeks have shown long-lasting effects, persisting for up to 5 weeks post-treatment (Frassinetti et al., 2002). These characteristics make PA a simple, non-invasive, and accessible method for inducing neuroplasticity within sensorimotor and cognitive networks (Clarke and Crottaz-Herbette, 2016; Tsujimoto et al., 2019).

The prismatic adaptation technique has emerged as a promising approach to enhancing calculation abilities and executive functions in individuals with dyscalculia by probably modulating the activity of specific cortical and subcortical regions. This technique induces a visuospatial error, which is corrected through a visuo-motor coordination task (pointing), activating a neural network involved in error processing and cognitive realignment (Pisella et al., 2006). Several studies have highlighted the neural mechanisms underlying Prismatic Adaptation (PA) in neurotypical individuals, reporting the activation of the temporoparietal cortex and cerebellum, and suggesting a spatial realignment between visual and proprioceptive systems maps (Pisella et al., 2006; Rempel-Clower et al., 1996). The rPA shifts the visual field to the right, enhances activation of the ventral attentional networks, facilitating a leftward shift of attention (Clarke and Crottaz-Herbette, 2016; Pisella et al., 2006). The contralateral occipital cortex is the first to register the visuospatial error, triggering an adaptive response that engages the cerebellum, which plays a key role in visuospatial realignment (Pisella et al., 2006). Subsequently, as we know thanks to previous studies, the temporal, frontal, and posterior parietal cortices are also recruited, contributing to multisensory integration, executive control, and numerical representation (Pisella et al., 2006). These areas are crucial for working memory, cognitive control, and numerical processing, and their activation overlaps with the regions typically impaired in dyscalculia, such as the intraparietal sulcus (IPS), inferior parietal lobule (IPL), and dorsolateral prefrontal cortex (DLPFC) (Mishra and Khan, 2023; Rotzer et al., 2009; McCaskey et al., 2020; Rykhlevskaia et al., 2009). Although PA is known to modulate activity in specific cortical and subcortical regions, the present study did not include neuroimaging measures. Therefore, any inference regarding underlying neurobiological mechanisms should be interpreted with caution.

Through this mechanism, prismatic adaptation may facilitate the improvement of numerical abilities, representing a novel intervention strategy for dyscalculia. PA relevance as a tool for cognitive and sensorimotor rehabilitation may consist of the potential to reorganize brain areas such as the posterior parietal cortex and cerebellum.

Moreover, the serious games incorporated in our tool are designed to stimulate attention, inhibitory control, and, most importantly, working memory and problem-solving skills. Specifically, our goal is to enhance higher-level cognitive processes to improve mathematical abilities.

Based on the results of the presented experimental study, it can be hypothesized that the combination of neurostimulation through prismatic adaptation and the selected serious games may represent an effective intervention for adolescents diagnosed with dyscalculia, engaging the same neural networks in different ways. Specifically, the initial stimulation with PA might serve as a booster for brain areas, which are then further trained through the serious games immediately afterward. Post-hoc within-group analyses indicate that both the control and experimental groups show a significant improvement over time, respectively with a small and a medium to large effect size. At the same time, there are no statistically significant differences between groups at baseline (T0), whether these differences become significant after the treatment (T1). This finding can be interpreted as evidence of the treatment’s effectiveness, beyond the improvement observed in the control group. We can hypothesize that our sample had cognitive abilities neither in decline nor in a stable condition but rather undergoing a gradual improvement. However, the treatment we proposed appears to significantly accelerate this process, and these substantial effect sizes may likely be driven by the inherent neural plasticity observed in adolescents aged 13–17 (Tymofiyeva and Gaschler, 2021; Park and Mackey, 2022; Velthuis and McAlonan, 2022).

Another possible explanation could be related to the differing levels of test–retest validity between memory and processing speed indices (Scharfen et al., 2018). In this case, our analyses may have captured the limitations of the assessment tool rather than a genuine improvement in the control group’s performance.

These findings highlight the potential of the digital rPA-cognitive treatment program as an effective and engaging intervention for adolescents with DD. The absence of clinical dropouts underscores the high compliance rate, suggesting that the program’s structure and design successfully maintain participant engagement. Notably, the integration of a child-friendly digital interface with embedded game elements enhances its accessibility, making it a promising tool for early DD remediation in younger children. Furthermore, the adaptive mechanism embedded in the training protocol ensures a personalized experience by continuously adjusting task difficulty based on individual performance. This dynamic adaptation not only optimizes cognitive challenge but also minimizes the risk of frustration, ultimately sustaining motivation throughout the intervention.

Timely intervention is a critical priority for children with developmental dyscalculia (Cheng et al., 2020), as difficulties in mathematical learning can significantly impact academic achievement (Xin et al., 2005), contribute to behavioral problems (Carballal Mariño et al., 2018), and increase the risk of school dropout (Alipanah et al., 2022; Lipka et al., 2019). Furthermore, challenges in school performance and wellbeing may negatively affect children’s psychological development (Benassi et al., 2022). Emerging evidence also highlights the neurobiological consequences of insufficient mathematical education. Adolescents who lack exposure to mathematical learning show reduced inhibitory control in key brain regions associated with reasoning and cognitive processing. This finding underscores the reciprocal relationship between brain development and education, demonstrating how the absence of specific educational experiences during adolescence can negatively impact neural plasticity and cognitive functions (Zacharopoulos et al., 2021).

Given the importance of early intervention during developmental stages, the availability of a non-invasive rehabilitation program that is time-efficient and capable of maintaining high engagement represents a valuable resource. Future studies should further investigate its longitudinal effects and explore potential refinements to maximize its impact on reading development.

### 4.1 Limitations

Several limitations of this study should be acknowledged. First, the sample was drawn exclusively from a single city in central Italy, which may limit the generalizability of the findings. Second, the relatively small sample size may limit the generalizability of the findings. Third, the waitlist control design controlling for some biases, does not fully rule out potential expectancy or placebo effects. Fourth, the absence of a long-term follow-up assessments prevents conclusions about the durability of the treatment effects. Moreover, the study did not include extended neuropsychological assessments, which could provide additional insights into the core numerical/arithmetical profiles of the participants.

Finally, in future studies, it could be interesting to include a physiological measure to observe the specific brain activity during the neurostimulation.

## 5 Conclusions and future directions

Our study highlights that the combination of PA and cognitive training effectively enhances cognitive skills and mathematical abilities in adolescents with DD. Consistent with our initial hypothesis, PA coupled with cognitive training appears to improve mathematical performance together with modulation of working memory and processing speed.

We suggest that the key to the effectiveness of this intervention lies precisely in the synergy between the two phases of training: an initial stimulation phase, where PA activates neural activity in brain regions involved in cognitive processing, followed by a targeted cognitive training phase aimed at strengthening the specific skills underlying mathematical deficits.

Our findings align with the *domain-general hypothesis*, demonstrating that the enhancement of attentional and executive functions (i.e., working memory, inhibition, and processing speed) plays a crucial role in improving calculation skills, including numerical fact retrieval and mental calculation. Moreover, this intervention effectively enhances both accuracy and response speed, providing a comprehensive approach to addressing mathematical difficulties. On the other hand, specific modulation of core numerical and arithmetical abilities associated with right hemispheric circuits cannot be excluded.

Finally, our results emphasize that a combined intervention—integrating neuromodulation with cognitive training—can be both time-efficient and engaging for adolescent patients while remaining highly effective. This study contributes to the growing body of research on intervention strategies for adolescents with DD, offering new insights into feasible and impactful treatment approaches.

Future studies should include extended follow-up periods, incorporate neuroimaging measures to directly assess neural mechanisms, and evaluate the generalization of training effects to real-world educational settings, including classroom performance.

## Data Availability

The raw data supporting the conclusions of this article will be made available by the authors, without undue reservation.

## Footnotes

1
www.restorativeneurotechnologies.com
