# Correction: Boosting executive functions and math abilities in adolescents with dyscalculia: the combined effect of prismatic adaptation and cognitive training

**DOI:** 10.3389/fpsyg.2026.1791373

**Published:** 2026-02-12

**Authors:** Lilian Zotti, Lauro Quadrana, Giulia Conte, Eleonora Sbardella, James Dawe, Agnese Di Garbo, Lina Pezzuti, Massimiliamo Oliveri

**Affiliations:** 1 Department of Dynamic and Clinical Psychology and Health, Sapienza University of Rome, Rome, Italy; 2 Department of Human Neurosciences, Sapienza University of Rome, Rome, Italy; 3 Department of Developmental and Social Psychology, Sapienza University of Rome, Rome, Italy; 4 Restorative Neurotechnologies Srl, Palermo, Italy; 5 Department of Biomedicine, Neurosciences and Advanced Diagnostics, University of Palermo, Palermo, Italy

**Keywords:** adolescence, cognitive training, dyscalculia, higher-order cognitive processes, prismatic adaptation

The figure captions were in the wrong order in the PDF and HTML versions of this paper.

Specifically, the captions for Figure 1 and Figure 2 were interchanged. The order has now been corrected.

The corrected caption of Figure 1 appears below.

“PA setup and timeline—modified with permission from (Bracco et al., 2018). Participants point to targets on a tablet screen (here depicted as curved surface, although the screen was flat). The dashed line marks the position on the screen of the visual target, while the arrow the pointing spot indicated by the participant. Pre-exposure (prismatic goggles off) involves pointing in free viewing conditions (both pointing movements and targets visible). During Exposure, participants wear the googles (rightward orientation) during free viewing pointing (exposure, goggles on). In post-exposure adaptation is then tested immediately after exposure with blinded pointing to targets (aftereffect).”

The corrected caption of Figure 2 appears below.

“CONSORT 2025 flow diagram of the progress through the phases of a randomised trial of two groups.”

A reference to Figure 1 was erroneously included in the wrong section.

A correction has been made to the Section 3 (Results), 3.2 (Main effects). This sentence previously stated: “Graphical representations are shown in Figure 1.” This sentence has been removed.

The original version of this article has been updated.

