# Correction: Archaea express circular isoforms of IS200/IS605-associated ωRNAs

**DOI:** 10.3389/fmicb.2026.1787636

**Published:** 2026-02-12

**Authors:** Beatriz A. Picinato, Vinícius H. Franceschini-Santos, Lívia S. Zaramela, Ricardo Z. N. Vêncio, Tie Koide

**Affiliations:** 1 Departamento de Bioquímica e Imunologia, Faculdade de Medicina de Ribeirão Preto, Universidade de São Paulo, Ribeirão Preto, Brazil; 2 Departamento de Computação e Matemática, Faculdade de Filosofia, Ciências e Letras, Universidade de São Paulo, Ribeirão Preto, Brazil

**Keywords:** circRNA, archaea, RNA-Seq, IS200/IS605, ωRNA, rRNA, tRNA

There was a mistake in Figure 4D as published. PK-2 sequence inside the box was displayed as “CACU” and the correct is “CAUU”. The corrected Figure 4D appears below.

**Figure 4:**
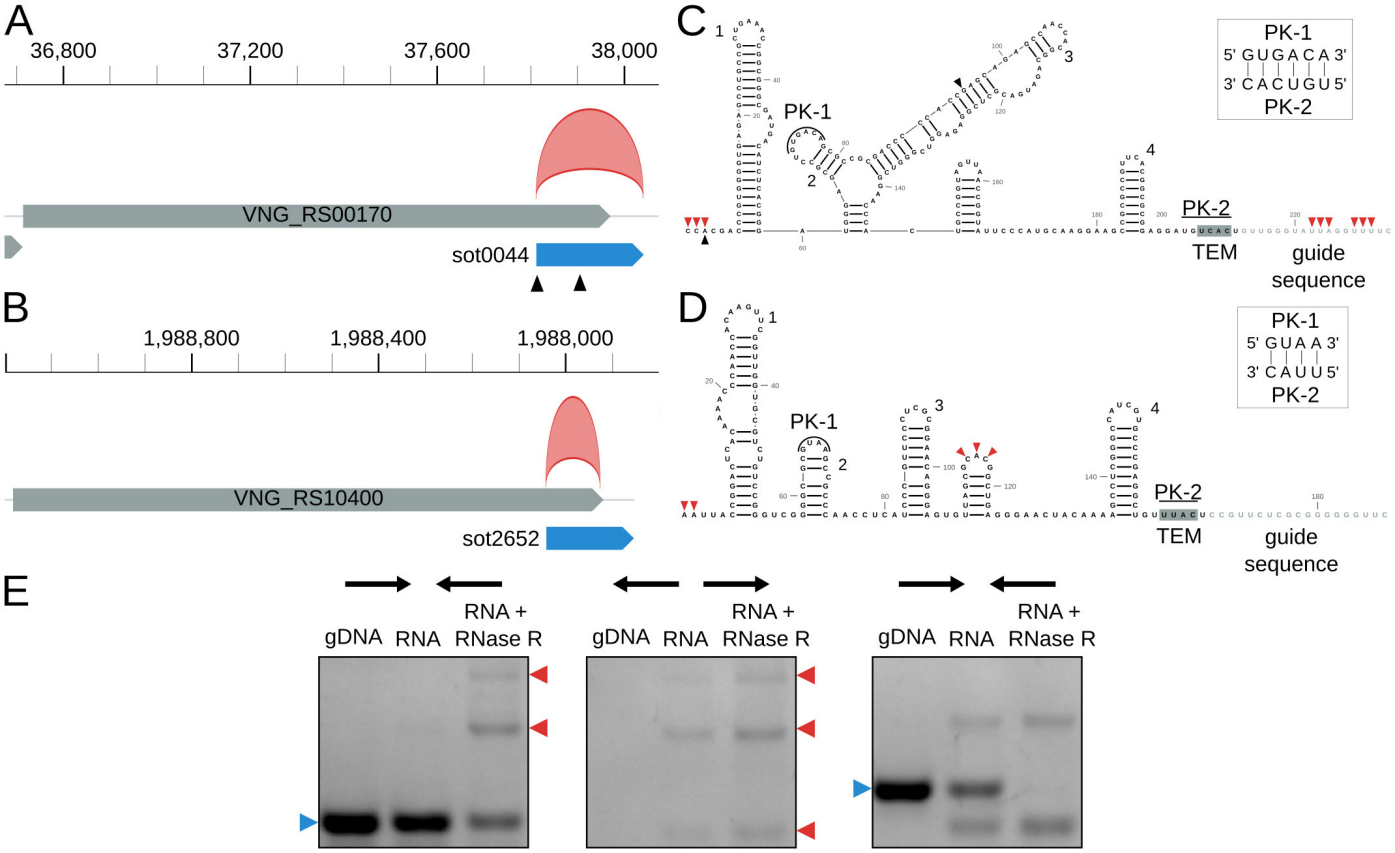
*Halobacterium salinarum* circRNAs in IS200/IS605 transposases and their ωRNAs. **(A)** VNG_RS00170/VNG0044H (gray box) and its ωRNA (blue box) with the annotated circRNA, circRNA_0012 (red arc). Black triangles mark transcript processing sites (TPS) associated with the ωRNA (Ibrahim et al., 2021). Coordinates on top of the main chromosome (NC_002607.1), in base pairs. **(B)** VNG_RS104000/VNG02652H (gray box) and its ωRNA (blue box) with the annotated circRNA, circRNA_0397 (red arc). Coordinates on top of the main chromosome (NC_002607.1), in base pairs. **(C)** RNA structure of sot0044 ωRNA. Black triangles mark the TPS as in panel A. Red triangles mark the start and end of junctions in circRNA_0012. Helices are numbered as in **Supplementary Figure S9**. **(D)** RNA structure of sot2652 ωRNA. Red triangles mark the start and end of junctions in circRNA_0012. Helices are numbered as in **Supplementary Figure S9**. PK, pseudoknot; TEM, transposon encoded motif. **(E)** RT-PCR validation of circRNA_0397 associated with sot2652. The left panel is a reaction made with convergent primers expected to amplify both linear and circular templates. The middle panel is the reaction made with divergent primers expected to amplify only circular products. The right panel is made with primers that amplify a linear product that is digested by RNAse R. Blue arrows (left) indicate the expected linear product, while red arrows (right) indicate the expected bands for circRNA junction amplification. The uncropped image of the agarose gel is in **Supplementary Figure S11**.

The funders Fundação de Apoio ao Ensino, Pesquisa e Assistência do Hospital das Clínicas da Faculdade de Medicina de Ribeirão Preto da Universidade de São Paulo (FAEPA) and FAPESP (São Paulo Research Foundation, Brazil), process 2025/22884-5 to Tie Koide were erroneously omitted.

The original version of this article has been updated.

