# Supplementary material for: Boosting executive functions and math abilities in adolescents with dyscalculia: the combined effect of prismatic adaptation and cognitive training
Source: Front Psychol. 2026 Jan 12;16:1669090. doi: 10.3389/fpsyg.2025.1669090 (PMC12833245; doi:10.3389/fpsyg.2025.1669090)
Supplement: Supplementary file 1 [file Supplementary_file_1.docx]

|  | Total | | | | | Treatment group | | | | | Control group | | | | | p |
| --- | --- | --- | --- | --- | --- | --- | --- | --- | --- | --- | --- | --- | --- | --- | --- | --- |
| Outcome | M (SD) | Min | Max | Skewness | Kurtosis | M (SD) | Min | Max | Skewness | Kurtosis | M (SD) | Min | Max | Skewness | Kurtosis |  |
| WMI | 79.29 (6.65) | 61 | 96 | -.33 | .565 | 77.80 (6.87) | 61 | 92 | -.30 | .235 | 80.77 (6.16) | 63 | 96 | -.265 | 1.307 |  |
| PSI | 78.63 (7.32) | 64 | 109 | 1.23 | 4.10 | 78.37 (8.40) | 64 | 109 | 1.67 | 5.207 | 78.89 (6.17) | 64 | 91 | .183 | .113 |  |
| MC speed | 1.90 (.725) | 1 | 3 | .155 | -1.06 | 1.91 (.742) | 1 | 3 | .14 | -1.11 | 1.89 (.72) | 1 | 3 | .174 | -.969 |  |
| MC accuracy | 1.99(.75) | 1 | 4 | .234 | -.59 | 1.97 (.822) | 1 | 4 | .39 | -.54 | 2.00 (.686) | 1 | 3 | .000 | -.749 |  |
| NF | 49.60 (8.07) | 22.66 | 59.30 | -1.11 | 1.55 | 49.33 (8.12) | 22.66 | 59.26 | -1.08 | 1.62 | 49.87 (8.12) | 22.70 | 59.30 | -1.198 | 1.927 |  |

**Table S1.** Descriptive statistics of outcome variables.

| Variable | Interaction | F | df | p | η² |
| --- | --- | --- | --- | --- | --- |
| WMI | Time*Group*Sex | .127 | 1,66 | 0.722 | 0.002 |
|  | Time*Group*Age | 1.495 | 1,66 | 0.226 | 0.024 |
| PSI | Time*Group*Sex | .016 | 1,66 | .900 | .000 |
|  | Time*Group*Age | 1.581 | 1,66 | .214 | .026 |
| MC Speed | Time*Group*Sex | .016 | 1,66 | 0.901 | .000 |
|  | Time*Group*Age | 1.669 | 1,66 | .201 | .027 |
| MC Accuracy | Time*Group*Sex | 2.793 | 1,66 | .100 | .043 |
|  | Time*Group*Age | .078 | 1,66 | .782 | .001 |
| NF | Time*Group*Sex | .001 | 1,66 | .973 | .000 |
|  | Time*Group*Age | .023 | 1,66 | .881 | .000 |

**Table S2.** Results from the interaction between time point, group (treatment vs control), and individual characteristics for each outcome variable.
